# Supplementary figures and images for: Laminar flow ventilation system to prevent airborne infection during exercise in the COVID-19 crisis: A single-center observational study
Source: PLoS One. 2021 Nov 10;16(11):e0257549. doi: 10.1371/journal.pone.0257549 (PMC8580245; doi:10.1371/journal.pone.0257549)

S1 Fig

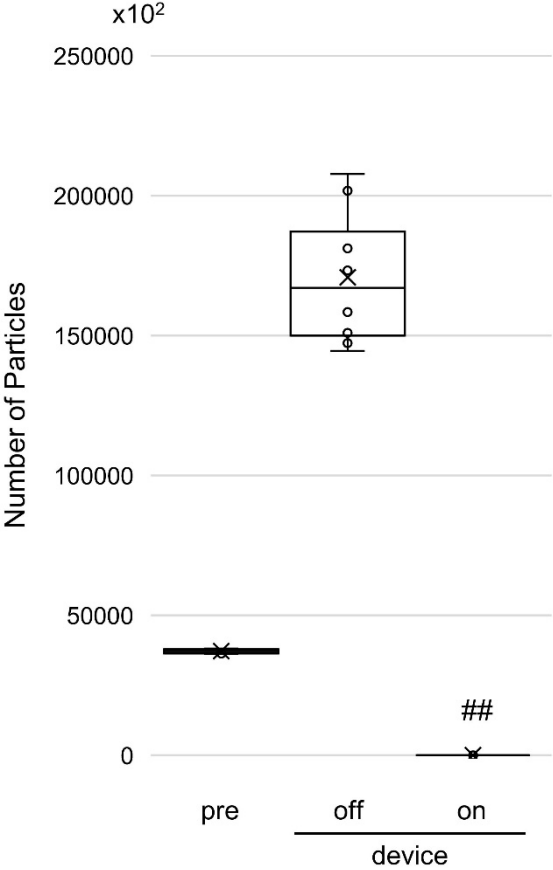

Supplement: S1 Fig — Particulates from an ultrasonic humidifier using the laminar airflow ventilation system (LFVS). The concentration of particulates (>0.3 μm) from an ultrasonic humidifier before and after the activation of the LFVS (n = 10). ##p<0.001 compared with the off LFVS. (PDF) [file pone.0257549.s001.pdf]
