# Supplementary material for: Laminar flow ventilation system to prevent airborne infection during exercise in the COVID-19 crisis: A single-center observational study
Source: PLoS One. 2021 Nov 10;16(11):e0257549. doi: 10.1371/journal.pone.0257549 (PMC8580245; doi:10.1371/journal.pone.0257549)
Supplement: Attachment — Submitted filename: Response_to_Reviewers_laminar.docx [file pone.0257549.s004.docx]

**KEIO UNIVERSITY SCHOOL OF MEDICINE**

DEPARTMENT OF CARDIOLOGY

35 SHINANOMACHI, SHINJUKU-KU

TOKYO 160-8582, JAPAN

TEL: +81-3-5843-6702

FAX: +81-3-5363-3875

E-MAIL: goodcentury21@keio.jp

**August 2, 2021**

James West, PhD

Academic Editor

*PLOS ONE*

Dear Editor:

Thank you for the critique of our manuscript, “Laminar flow ventilation system to prevent airborne infection during exercise in the COVID-19 crisis: a single-center observational study” (PONE-D-21-09706). We acknowledge the concerns raised by the reviewer and thank them for their time and effort. Their comments have helped us to restructure the revised manuscript, which we now submit for consideration for publication. Please find the revised manuscript with a point-by-point response to the reviewers’ comments. The laminar airflow ventilation system was developed in collaboration with E.K. and Nippon Medical & Chemical Instruments Co. Ltd. This work was partly supported by a Grant-in-Aid from Scientific Research from the Japan Agency for Medical Research and Development [ID. 21ek0210130h0003] and by Kimura Memorial Heart Foundation Research Grant for 2019 [N/A], Suzuken Memorial Foundation [N/A], Foundation for Total Health Promotion [N/A], and Research Grant for Public Health Science [N/A].The funders provided support in the form of financial supports for authors [Y.K., E.K.], but did not have any additional role in the study design, data collection and analysis, decision to publish, or preparation of the manuscript.

Sincerely,

Yoshinori Katsumata, MD, PhD

Department of Cardiology, Keio University School of Medicine

35 Shinanomachi Shinjuku-ku, Tokyo, Japan 160-8582

Tel: +81-3-3353-1211

Fax: +81-3-5363-3875

E-mail: goodcentury21@keio.jp

**To Reviewer #1**

#1. I think it is a very valuable paper especially in regard to present situation. Though I would be very happy if you could perform Power analysis (then you do not have to say in limitations that you have a small sample)

**Response:**

- Thank you very much for pointing this out. As suggested, a power analysis was performed. Based on a pre-performed Shapiro-wilk test, multiple comparisons of changes (Δ) in the number of airborne particulates involving each incremental exercise period from the warm-up were made using the repeated analysis of variance with the Dunnet test as a post-hoc test. It revealed a significant and gradual increase in the respiratory droplets with time with a large effect size. Also, Cohen’s d was calculated using the value of t for paired t test to compare the droplet concentrations from spray or from the oral cavity during vigorous exercise before and after the activation of LFVS. We revised the related parts of the *Materials and Methods, Results, Discussion, and Table*, as shown below:

*-Method section (Statistical analyses)-*

*The results are presented as means with standard deviations for continuous variables and as percentages for categorical variables, as appropriate. Based on a pre-performed Shapiro-wilk test, multiple comparisons of changes (Δ) in the number of airborne particulates involving each incremental exercise period from the warm-up were made using the repeated analysis of variance with the Dunnet test as a post-hoc test~~Steel test~~. Student’s paired t-test was used* *to compare the droplet concentrations from spray or from the oral cavity during vigorous exercise before and after the activation of LFVS.* *We calculated Cohen’s d using the value of t for paired t test. SPSS, version 25.0 (SPSS Inc., Chicago, Illinois), was used for analysis, and p<0.05 (2-sided) was set to define statistical significance.*

*-Result section-*

***Monitoring particulates during exercise***

*Baseline characteristics of the subjects are summarized in Table 1. The subjects were predominantly male (80%), with an average age of 29 ± 12 years. Figure 3 shows the representative data for the particulates, lactate in sweat, and heart rate during exercise. A steady number of particulates before the LT was followed by a significant and gradual increase in the respiratory droplets after the LT, particularly during anaerobic exercise* *with a large effect size (Fig. 3 and Table 2).*

*Table2. Airborne Particulate Generation During Exercise.*

|  | *rest* | *Warm-up* | *Pre-LT* | *Post-LT* | *85%_1* | *85%_2* |
| --- | --- | --- | --- | --- | --- | --- |
| *Mean difference ± SD  (95%CI)* | *731.3 ± 760.0 (-2727.6 - 4190.2)* | *-* | *-27.5 ± 850.9 (-3591.9 - 3537.0)* | *1552.4 ± 1667.4 (-2012.0 - 5116.9)* | *4653.4 ± 3380.5 (1088.9 - 8217.8)* | *10727.3 ± 7060.6 (7162.8 ± 14291.7)* |
| *p-value^#^* | *0.97* | *-* | *1.00* | *0.64* | *< 0.01* | *< 0.001* |
| *Power* | *0.77* | *-* | *0.05* | *0.75* | *1.00* | *1.00* |

*Repeated analysis of variance with the Dunnet test as a post-hoc test revealed a significant and gradual increase in the respiratory droplets with time. ^#^p value compared with the warm-up. LT, lactate threshold; 85%_1, the first half of the constant-load exercise at 85% of the expected maximum heart rate; 85%_2, the latter half of the constant-load exercise at 85% of the expected maximum heart rate.*

*-Result section-*

***Effect of the LFVS on the particulates during vigorous exercise***

*The LFVS was activated during a constant load of exercise intensity above the LT, in which the concentration of particulates in the exhaled air increased. Notably, particulates exhaled during exercise were almost completely removed by the LFVS (>0.3 μm: 2120800±759700 vs. 560 ± 170; p<0.001, p<0.001, n=10; Fig. 4 and Table 3).*

*Table 3. Airborne Particulate Generation During Anaerobic Exercise Using the Laminar Airflow Ventilation System (LFVS).*

|  | *LFVS*  *Off* | *LFVS*  *On* | *Difference* | | *p-value* | *Cohen's d* | | *Power* |
| --- | --- | --- | --- | --- | --- | --- | --- | --- |
|  |  |  | *Mean (SD)* | *95%CI* |  | *Point* | *95%CI* |  |
| *Number of particles* | *21208.2  (7596.9)* | *5.6  (1.7)* | *21202.7  (7596.9)* | *15768.2 - 26637.1* | *p < 0.01* | *2.791* | *1.368 - 4.188* | *1.00* |

*Mean (SD) was shown.*

*Abbreviation: LFVS, Laminar airflow ventilation system*

*-Discussion section-*

***Limitations***

*The present study has some limitations. First, as a single-center observational study, there may have been a selection bias. Second, ~~the number of subjects was small. Third,~~ our study did not allow for an examination of the possibility of LFVS in preventing the COVID-19 infection. A further randomized study with a large sample size and long-term follow-up for the prevention of COVID-19 infection is required to overcome these limitations.*

#2. Also regarding the language. Do not use WE did this, WE did that. Please use impersonal, passive voice.

**Response:**

- As you mentioned, we revised the manuscript to remove the use of “We” when not appropriate.

Journal Requirements:

When submitting your revision, we need you to address these additional requirements.

1. Please ensure that your manuscript meets PLOS ONE's style requirements, including those for file naming. The PLOS ONE style templates can be found at

<https://journals.plos.org/plosone/s/file?id=wjVg/PLOSOne_formatting_sample_main_body.pdf> and

<https://journals.plos.org/plosone/s/file?id=ba62/PLOSOne_formatting_sample_title_authors_affiliations.pdf>

**Response:**

- As suggested, we revised the manuscript in accordance with PLOS ONE's style requirements.

2. In your Methods section, please provide additional information about the participant recruitment method and the demographic details of your participants. Please ensure you have provided sufficient details to replicate the analyses such as: a) the recruitment date range (month and year), b) a description of any inclusion/exclusion criteria that were applied to participant recruitment, c) a statement as to whether your sample can be considered representative of a larger population, d) a description of how participants were recruited."

3. During the internal evaluation of the manuscript we have noted in the Methods section that one patient experienced a subdural hematoma. Please provide further clarification regarding whether this was a direct result of the exercise intervention. Please provide details regarding any treatment which the participant received.

**Response:**

- As suggested, we have revised the Methods section, as shown below:

*-Method Section-*

*Subjects aged 20-80 years were recruited via a web system in October 2020. Exclusion criteria included receiving medication and having comorbidities, such as hypertension, diabetes, or active lung diseases. Twenty healthy subjects ~~were~~ enrolled ~~recruited~~ in this study ~~The subjects~~ had a broad spectrum of aerobic capacities and fitness levels, including athletes~~, and had no comorbidities, such as hypertension, diabetes, or active lung diseases~~. One of them had experienced a subdural hematoma 6 months ago. At the time he participated in this study, he was cured of his disease and was not receiving ongoing treatment. In addition, he was actively engaged in daily exercise. All of them received no medication. They can be considered representative of a larger population. The study protocol was approved by the Institutional Review Board (IRB) of Keio University School of Medicine [permission number; 20190229], and the study was conducted in accordance with the Declaration of Helsinki. Subjects provided verbal informed consent, because* *the IRB approved use of oral consent in accordance with Japanese guidance for clinical research. Verbal consents were recorded as experimental notes in this study.*

4. Please provide additional details regarding participant consent. In the ethics statement in the Methods and online submission information, please ensure that you have specified: 1) whether the ethics committee approved the verbal/oral consent procedure, 2) why written consent could not be obtained, and 3) how verbal/oral consent was recorded. If your study included minors, please state whether you obtained consent from parents or guardians in these cases. If the need for consent was waived by the ethics committee, please include this information.

**Response:**

- Thank you very much for pointing this out. The IRB approved the use of oral consent in accordance with Japanese guidance for clinical research. The verbal consent was recorded as an experimental note in this study. Subjects aged 20-80 years old were recruited. We revised the Methods section, as shown below:

*-Method Section-*

*Subjects aged 20-80 years were recruited via a web system in October 2020. Exclusion criteria included receiving medication and having comorbidities, such as hypertension, diabetes, or active lung diseases. Twenty healthy subjects ~~were~~ enrolled ~~recruited~~ in this study ~~The subjects~~ had a broad spectrum of aerobic capacities and fitness levels, including athletes~~, and had no comorbidities, such as hypertension, diabetes, or active lung diseases~~. One of them had experienced a subdural hematoma 6 months ago. At the time he participated in this study, he was cured of his disease and was not receiving ongoing treatment. In addition, he was actively engaged in daily exercise. All of them received no medication. They can be considered representative of a larger population. The study protocol was approved by the Institutional Review Board (IRB) of Keio University School of Medicine [permission number; 20190229], and the study was conducted in accordance with the Declaration of Helsinki. Subjects provided verbal informed consent because the IRB approved use of oral consent in accordance with Japanese guidance for clinical research. Verbal consents were recorded as experimental notes in this study.*

5. Thank you for providing the following Funding Statement:

"Yoshinori Katsumata has a financial relationship with Kimura Memorial Heart Foundation Research Grant for 2019, Suzuken Memorial Foundation, Foundation for Total Health Promotion, and Research Grant for Public Health Science. Eiji Kobayashi has a financial relationship with Nippon Medical & Chemical Instruments Co. Ltd. Motoaki Sano, Hiroki Okawara, Tomonori Sawada, Genki Ichihara, and Kazuki Sato declare that they have no competing interests."

We note that one or more of the authors is affiliated with the funding organization, indicating the funder may have had some role in the design, data collection, analysis or preparation of your manuscript for publication; in other words, the funder played an indirect role through the participation of the co-authors.

If the funding organization did not play a role in the study design, data collection and analysis, decision to publish, or preparation of the manuscript and only provided financial support in the form of authors' salaries and/or research materials, please review your statements relating to the author contributions, and ensure you have specifically and accurately indicated the role(s) that these authors had in your study in the Author Contributions section of the online submission form. Please make any necessary amendments directly within this section of the online submission form.  Please also update your Funding Statement to include the following statement: “The funder provided support in the form of salaries for authors [insert relevant initials], but did not have any additional role in the study design, data collection and analysis, decision to publish, or preparation of the manuscript. The specific roles of these authors are articulated in the ‘author contributions’ section.”

If the funding organization did have an additional role, please state and explain that role within your Funding Statement.

Please also provide an updated Competing Interests Statement declaring this commercial affiliation along with any other relevant declarations relating to employment, consultancy, patents, products in development, or marketed products, etc.

Within your Competing Interests Statement, please confirm that this commercial affiliation does not alter your adherence to all PLOS ONE policies on sharing data and materials by including the following statement: "This does not alter our adherence to PLOS ONE policies on sharing data and materials.” (as detailed online in our guide for authors <http://journals.plos.org/plosone/s/competing-interests>). If this adherence statement is not accurate and there are restrictions on sharing of data and/or materials, please state these. Please note that we cannot proceed with consideration of your article until this information has been declared.

**Response:**

- As suggested, we revised the Methods section, as shown below:

*Funding Statement*

*The laminar airflow ventilation system was developed in collaboration with E.K. and Nippon Medical & Chemical Instruments Co. Ltd. This work was partly supported by a Grant-in-Aid from Scientific Research from the Japan Agency for Medical Research and Development [ID. 21ek0210130h0003] and by Kimura Memorial Heart Foundation Research Grant for 2019 [N/A], Suzuken Memorial Foundation [N/A], Foundation for Total Health Promotion [N/A], and Research Grant for Public Health Science [N/A].*

*The funders provided support in the form of financial supports for authors [Y.K., E.K.], but did not have any additional role in the study design, data collection and analysis, decision to publish, or preparation of the manuscript. The specific roles of these authors are articulated in the ‘author contributions’ section.*

*Competing Interests Statement*

*Yoshinori Katsumata has a financial relationship with Kimura Memorial Heart Foundation Research Grant for 2019, Suzuken Memorial Foundation, Foundation for Total Health Promotion, and Research Grant for Public Health Science. Eiji Kobayashi has a financial relationship with Nippon Medical & Chemical Instruments Co. Ltd. Motoaki Sano, Hiroki Okawara, Tomonori Sawada, Genki Ichihara, and Kazuki Sato declare that they have no competing interests.*

*This does not alter our adherence to PLOS ONE policies on sharing data and materials.*

6. In your Data Availability statement, you have not specified where the minimal data set underlying the results described in your manuscript can be found. PLOS defines a study's minimal data set as the underlying data used to reach the conclusions drawn in the manuscript and any additional data required to replicate the reported study findings in their entirety. All PLOS journals require that the minimal data set be made fully available. For more information about our data policy, please see <http://journals.plos.org/plosone/s/data-availability>.

"Upon re-submitting your revised manuscript, please upload your study’s minimal underlying data set as either Supporting Information files or to a stable, public repository and include the relevant URLs, DOIs, or accession numbers within your revised cover letter. For a list of acceptable repositories, please see <http://journals.plos.org/plosone/s/data-availability#loc-recommended-repositories>. Any potentially identifying patient information must be fully anonymized.

Important: If there are ethical or legal restrictions to sharing your data publicly, please explain these restrictions in detail. Please see our guidelines for more information on what we consider unacceptable restrictions to publicly sharing data: <http://journals.plos.org/plosone/s/data-availability#loc-unacceptable-data-access-restrictions>. Note that it is not acceptable for the authors to be the sole named individuals responsible for ensuring data access.

We will update your Data Availability statement to reflect the information you provide in your cover letter.

**Response:**

- As suggested, we uploaded the minimal data set. And, we revised the manuscriot, as shown below:

***Availability of data and material***

*The datasets* are within the manuscript and its Supporting Information files.

*~~The datasets generated during and/or analyzed during the current study are not publicly available but are available from the corresponding author on reasonable request.~~*

7. Your ethics statement should only appear in the Methods section of your manuscript. If your ethics statement is written in any section besides the Methods, please delete it from any other section.

**Response:**

- As suggested, we revised the manuscript.

8. We note that Figure 1 in your submission contain copyrighted images. All PLOS content is published under the Creative Commons Attribution License (CC BY 4.0), which means that the manuscript, images, and Supporting Information files will be freely available online, and any third party is permitted to access, download, copy, distribute, and use these materials in any way, even commercially, with proper attribution. For more information, see our copyright guidelines: <http://journals.plos.org/plosone/s/licenses-and-copyright>.

We require you to either (1) present written permission from the copyright holder to publish these figures specifically under the CC BY 4.0 license, or (2) remove the figures from your submission:

1. You may seek permission from the original copyright holder of Figure 1 to publish the content specifically under the CC BY 4.0 license.

We recommend that you contact the original copyright holder with the Content Permission Form (<http://journals.plos.org/plosone/s/file?id=7c09/content-permission-form.pdf>) and the following text: “I request permission for the open-access journal PLOS ONE to publish XXX under the Creative Commons Attribution License (CCAL) CC BY 4.0 (<http://creativecommons.org/licenses/by/4.0/>). Please be aware that this license allows unrestricted use and distribution, even commercially, by third parties. Please reply and provide explicit written permission to publish XXX under a CC BY license and complete the attached form.”

Please upload the completed Content Permission Form or other proof of granted permissions as an "Other" file with your submission. In the figure caption of the copyrighted figure, please include the following text: “Reprinted from [ref] under a CC BY license, with permission from [name of publisher], original copyright [original copyright year].”

2. If you are unable to obtain permission from the original copyright holder to publish these figures under the CC BY 4.0 license or if the copyright holder’s requirements are incompatible with the CC BY 4.0 license, please either i) remove the figure or ii) supply a replacement figure that complies with the CC BY 4.0 license. Please check copyright information on all replacement figures and update the figure caption with source information. If applicable, please specify in the figure caption text when a figure is similar but not identical to the original image and is therefore for illustrative purposes only.

**Response:**

- As suggested, we completed the Content Permission Form.

9. Please review your reference list to ensure that it is complete and correct. If you have cited papers that have been retracted, please include the rationale for doing so in the manuscript text, or remove these references and replace them with relevant current references. Any changes to the reference list should be mentioned in the rebuttal letter that accompanies your revised manuscript. If you need to cite a retracted article, indicate the article’s retracted status in the References list and also include a citation and full reference for the retraction notice.

**Response:**

- Thank you. We have not cited papers that have been retracted. No changes were made to the references.
